# Experimental Exploration of Objective Human Pain Assessment Using Multimodal Sensing Signals

**DOI:** 10.3389/fnins.2022.831627

**Published:** 2022-02-11

**Authors:** Yingzi Lin, Yan Xiao, Li Wang, Yikang Guo, Wenchao Zhu, Biren Dalip, Sagar Kamarthi, Kristin L. Schreiber, Robert R. Edwards, Richard D. Urman

**Affiliations:** ^1^Intelligent Human Machine Systems Laboratory, College of Engineering, Northeastern University, Boston, MA, United States; ^2^College of Nursing and Health Innovation, University of Texas at Arlington, Arlington, TX, United States; ^3^Department of Anesthesiology, Perioperative and Pain Medicine, Brigham and Women’s Hospital, Harvard University, Boston, MA, United States

**Keywords:** pain measurement, sensors, physiological signals, machine learning algorithms, multi-modality sensor fusion

## Abstract

Optimization of pain assessment and treatment is an active area of research in healthcare. The purpose of this research is to create an objective pain intensity estimation system based on multimodal sensing signals through experimental studies. Twenty eight healthy subjects were recruited at Northeastern University. Nine physiological modalities were utilized in this research, namely facial expressions (FE), electroencephalography (EEG), eye movement (EM), skin conductance (SC), and blood volume pulse (BVP), electromyography (EMG), respiration rate (RR), skin temperature (ST), blood pressure (BP). Statistical analysis and machine learning algorithms were deployed to analyze the physiological data. FE, EEG, SC, BVP, and BP proved to be able to detect different pain states from healthy subjects. Multi-modalities proved to be promising in detecting different levels of painful states. A decision-level multi-modal fusion also proved to be efficient and accurate in classifying painful states.

## Introduction

Optimization of pain assessment and treatment is an ongoing area of research in healthcare (Pathak et al., 2018; Hohenschurz-Schmidt et al., 2020). Verbal or graphic scales for patients being evaluated for acute or chronic pain are not usually adequate. It is well established that different person’s experience pain differently, and their communication of their pain intensity on the verbal scale does not easily allow healthcare providers to gauge the persons’ pain level correctly and treat them effectively.

Providers commonly use the numeric rating scale (NRS) for pain assessment and treat the patients on the assumption that the pain assessments are accurate (McCaffery and Beebe, 1989). In a clinical setting, patients are asked to provide a verbal description of their pain intensity on a 0–10 scale. Zero (0) being no pain and 10 being severe pain. Tandon et al. (2016) conducted a study to validate a 4-point objective pain score (OPS) to evaluate acute postoperative pain and compare it with the standard NRS. They studied a total of 1,021 paired readings of the two methods (OPS and NRS) in 93 patients who underwent a laparotomy and used patient-controlled analgesia. They also concluded that the OPS is desirable as it can work independently on its own as well as alongside NRS.

The side effects of opioids are an issue and are often brought about after patients have been treated for chronic pain. However, the rate of recurrence of opioid side effects has not often been observed when dealing with acute pain. As reported in Daoust et al. (2020), short-term incidence of opioid-induced side effects were evaluated, which include constipation, nausea/vomiting, dizziness, drowsiness, sweating, and weakness in patients discharged from the emergency department (ED) with an opioid prescription. The results showed that oral opioid side effects are common during the short-term care of acute pain in ED discharged patients. There are many factors to consider in a pain study; they include age, sex, preexisting health conditions, previous injuries, and health conditions that the patient may be oblivious. Given that it is not possible for healthcare providers to know the exact amount of pain that patients feel, they use their best judgment. To optimize the accuracy of clinical treatment by eliminating the patients’ subjectivity and minimize the risks of putting patients in a position where they may have to apply “guesswork,” bio-sensors are utilized and algorithms are developed to obtain a continuous measurement of physiological parameters for pain measurements in real-time will drastically ease the time and workload for healthcare providers (Lin et al., 2018; Wang et al., 2020, 2021; Yu et al., 2020a,b; Guo et al., 2021).

The clinical symptoms associated with acute pain frequently include increased heart rate, blood pressure, respiratory rate, shallow respiration, agitation, restlessness, facial grimace, and splinting. The approach of patients reporting their own pain levels to the caregiver is highly subjective, and often inaccurate. Algorithms for objective measurement will increase accuracy and allow healthcare providers to work with ease and efficiency. These algorithms can utilize bio-sensors and wearable devices to receive data from the patient (Stern et al., 2006; Kächele et al., 2016; Yang et al., 2017). Bio-potentials, the electric potentials that transfer information between living cells, can be measured and processed using electrocardiography (ECG), electroencephalography (EEG), or electromyography (EMG), to observe nociception and pain. When an individual’s nociceptive threshold is met, the nociceptive flexion reflex assesses the protective withdrawal reflex and provides a signal (Cowen et al., 2015).

Campbell et al. (2019) used BioVid heat pain dataset to extract a total of 155 features from three signals namely ECG, EMG, and SC. The 155 features are related to seven properties of the signal. The seven properties are: to (i) variability, (ii) similarity, (iii) linearity, (iv) stationarity, (v) amplitude, (vi) entropy, and (vii) frequency. They then used a topologically informed chart called Mapper to visualize associations between these features. The purpose of the work was to address the variability in the optimal set of features used in related literature. Chu et al. (2017) extracted 36 features from three signal modalities and then used the genetic algorithm for feature selection and principal component analysis (PCA) for feature reduction. Following that they used LDA, KNN, and SVM to classify the pain intensity.

As pain sensation levels vary between individuals, Patient A may experience very little pain while Patient B may experience a lot of pain when equal pain intensity is being applied. In their study of automatic pain quantification using autonomic parameters, Walter et al. (2014) showed this to be a significant phenomenon impacting both clinical care and pain research. The authors gathered study participants who underwent a heat stimulus test to demonstrate this variability in response. Eighty-six participants between the ages of 18–35 participated in this study. The EMG and ECG were the measured parameters for this experiment. Participants were seated and a PATHWAY thermode (produced by Medoc, Israel) was applied to their right arms. This thermode was used by the researchers to apply a controlled direct heat under 50.5°C – to avoid skin burns. The most selective features they used were the EMG corrugator peak-to-peak, corrugator Shannon entropy, and heart rate variability slope RR (Walter et al., 2014). RR is the interval between successive R’s measured from the peak of the QRS complex of the ECG wave. Walter et al. (2014) found that the EMG features significantly added to the quantification of pain. From their statistical analysis, they concluded that a general feature pattern was detected, but detection rates can be significantly improved through individual-specific calibration.

There are various tools and methods for observing pain levels in unconscious patients or those that simply cannot comprehend and communicate their pain. For example, Kanji et al. (2016) designed a prospective cohort study to test a validated pain assessment tool, the Critical Care Pain Observation Tool (CPOT). This tool was used to study patients with signs of delirium. The CPOT included facial expression, body movements, muscle tension, and compliance with a ventilator or vocalization (Bokoch et al., 2015).

There are opportunities to develop and validate non-intrusive methods of measuring objective pain measurements. For example, when patients are in critical conditions, they may not be able to communicate their discomfort effectively to their healthcare providers. With bio-sensors, pain can be assessed with little to no movement from patients. This is important because patients suffering from acute pain may not be able to move, and their pain levels may increase if they try, and the constant changing of pain levels may lead to variation in pain assessment. If they communicate “sometimes it feels like a 3, sometimes it feels like a 10 on a 0–10 scale,” then the healthcare providers will have to guess the appropriate treatment and medication dosages. A person’s pain perception and consequent self-rating can be controlled by coping mechanisms such as deep breathing, meditation, or affected by previous experiences with pain and pain tolerance. However, bio-sensors can accurately record and interpret the pain. Designing bio-sensors and algorithms for objective pain measurements will greatly benefit the healthcare industry.

To help the clinicians get continuous and objective pain assessment results, and to improve the accuracy of the pain assessment by supplementing the traditional verbal scale, the Continuous Objective Multimodal Pain Assessment Sensing System (COMPASS) study was carried out which contains nine modalities: (1) facial expressions (FE), (2) electroencephalography (EEG), (3) eye movement (EM), (4) skin conductance (SC), (5) blood volume pulse (BVP), (6) electromyography (EMG), (7) respiration rate (RR), (8) skin temperature (ST), and (9) blood pressure (BP). In the following sections, the nine modalities and the fusion of them are discussed. The aim of the COMPASS is to support clinicians in their initial pain measurements. If measurements from a single bio-sensor turn out to be not statistically significant, clinicians can incorporate more bio-sensors into their practice to increase the accuracy of their observations. This technology could provide clinicians with an efficient way of assessing pain levels and administering treatments safely and accordingly.

The remaining of the manuscript is organized as follows: Section “Materials and Methods” describes the materials and methods of the study. Section “Results” shows the key experiment results. Section “Discussion” discusses the findings of the present study and next steps to further investigation including potential clinical applications. Section “Conclusion” concludes the manuscript.

## Materials and Methods

### Subjects

A total of 28 healthy subjects were recruited in this research at Northeastern University. Among them, 26 finished three repeats of the experiment. Our data set included seven male subjects and 19 female subjects (average age = 20.7 years, SD = 2.4). None of the subjects reported any kinds of pain before the experiment.

### Apparatus

Enobio 32 (produced by Neuroelectrics, Spain) is a wireless sensor and has 500 Hz sampling rates, which can get sufficient information. It was used to collect EEG signal. Tobii Pro Glasses 2 (produced by Tobii, Sweden) is a mobile eye tracing sensor with four eye tracking cameras. It was used to collect the pupillary response data due to its high performance of eye tracking. FlexComp Infiniti (produced by Thought Technology, Canada) is a physiological monitoring and data acquisition sensor and can collect several physiological data at the same time. It was used to collect the skin conductance, electromyography, blood volume pulse, skin temperature, and respiration rate. An Omron series 5 (Omron, Japan) was used to collect the blood pressure signal. A built-in web camera from a Dell laptop was used to capture the facial expression images. A verbal rating scale was used to record the subjective ratings reported by the subjects. A Dell desktop was used to run the data analysis.

### Procedure

**FIGURE 1:**
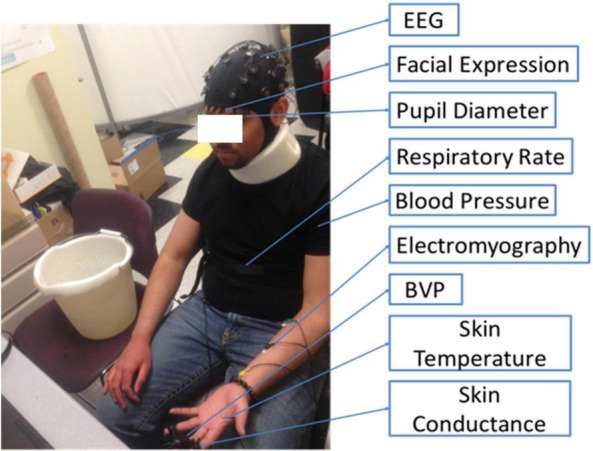
The experimental set up of the present pain assessment study at the Intelligent Human-Machine Systems Lab at Northeastern University. The locations of nine sensors attached to a participant are marked.

**FIGURE 2:**
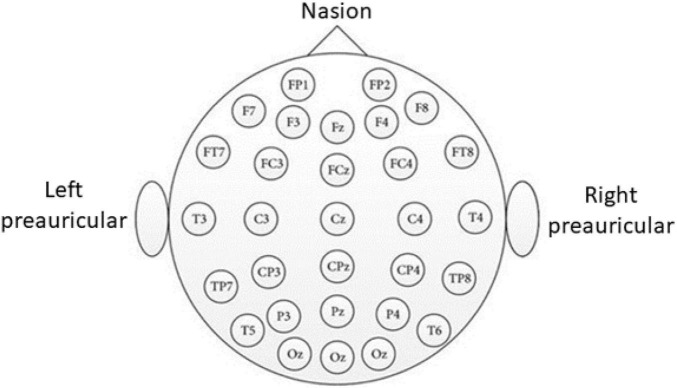
The 10–20 international system used for EEG channel location. (1) Distances between adjacent electrodes being 10 or 20% of the total front-back or left to right distance along the skull, (2) Each site is named a letter and a number, (3) Letters F, T, C, P, and O identify the Frontal, Central, Parietal, and Occipital lobes, respectively, (4) The numbers identify the location along the hemisphere where even numbers are to the right and odd numbers are to the left, and (5) the letter Z identifies the electrode placed on the midline.

The study protocol was approved by Northeastern University institutional review board (IRB), IRB #17-01-25. All subjects were from Northeastern University recruited through flier and social media advertisement and were provided written consent before starting the experiment. The experiment was repeated three times in 3 days of three different weeks for each subject to get sufficient data and to study if there were any differences between different days. The experimental setting is shown in Figure 1. The experimental procedures are as follows:

(1) Sensors were set up and a recording camera was mounted in front of the participant, at a distance of 30 cm.
  (a) EEG was set up on the subject’s scalp, using the 10–20 system, as shown in Figure 2.
  (b) BVP was taken using FlexComp Infiniti BVP sensor placed on the subject’s middle finger.
  (c) Skin conductance was taken using FlexComp Infiniti skin conductance sensor placed on the subject’s ring and index fingers.
  (d) EMG measures muscle response or electrical activity in response to a nerve’s stimulation of the muscle, which was taken using the FlexComp Infiniti EMG sensor placed on the subject’s left forearm.
  (e) Skin Temperature was taken using FlexComp Infiniti skin temperature sensor (non-intrusive electrode) attached to the dorsal aspect of the subject’s left hand.
  (f) Respiration Rate was taken using FlexComp Infiniti respiration rate belt placed around the circumference of the subject’s stomach.
  (g) Pupillary diameter was taken using Tobii glasses eye-tracker.
  (h) Blood pressure was taken from the subject’s left arm using Omron series 5.
(2) The subject was asked to maintain focus on a green dot displayed on a monitor in front of him/her.
(3) The subject was asked to relax and a 20-s recording of all sensors was taken in the relaxed state as a baseline measurement.
(4) The subject was asked to put his/her hand into iced water until the experiment ended or when the subject told us he or she cannot bear the pain.
(5) Every session (20 s), the subject was asked for his/her pain level from 0 to 10, using the verbal rating scale (VRS), in which 0 meant no pain and 10 meant most painful.
(6) The experiment ended after 10 sessions or any time the subject wanted to stop.
(7) Once the experiment was over, the subject was asked to complete a survey with questions pertaining to the pain experienced during the experiment.

### Data Description and Pre-processing

Table 1 shows a summary of all the sensors utilized in this research. The summary includes a brief description of the sensor, the collection devices, and the sampling rate of each sensor. The sampling rate of the web camera, the Enobio, the Tobii Glasses Pro, the Thought Technology, and the Omran was 30, 500, 50, and 2,048 Hz, respectively.

**TABLE 1:** A summary of all the sensors (description, devices, and sampling rate).

Sensor	Description	Device	Sampling rate
FE	The facial expression of the subjects	Web camera	30 Hz
EEG	The brain wave of the subjects	Enobio 32	500 Hz
EM	Diameter of human eyes	Tobii Glasses Pro	50 Hz
SC	Conductivity of skin	Thought technology	2,048 Hz
BVP	Blood that passes through the tissues	Thought technology	2,048 Hz
EMG	Muscle activities	Thought technology	2,048 Hz
ST	Temperature on skin	Thought technology	2,048 Hz
RR	Number of respiration per minute	Thought technology	2,048 Hz
BP	Number of respiration per minute	Omron series 5	NA

Facial expression-based pain estimation is a key part of our system because it is real-time, convenient, non-intrusive, and automatic. Because the videos we collected contained other parts of the subjects’ body as well as some backgrounds that we didn’t use, we decided to use face region detection. Since our goal is to develop a real-time pain estimation system, we didn’t apply the common face detection algorithm R-CNN (Girshick et al., 2014) because it is computationally heavy. Instead, the Adaboost (Schapire, 2013) algorithm is used as our face detection method because it performs faster and can be easily applied to the real-time scenario.

The next step is feature extraction step. In this step, we use the facial action unit analysis. Action units (AUs) are the fundamental actions of an individual that appears in the facial expression and constitute the Facial Action Coding System (FACS). In our study, we utilize the Openface 2.0 (Baltrusaitis et al., 2018) as our tool to get the probabilities of AUs as the facial features. The output of the Openface 2.0 is the probabilities of the 17 AUs. The details are shown in Table 2. All the AUs are obtained through a Convolutional Neural Network based face detector and facial landmark detection algorithm. The reason why we chose the Openface 2.0 is that it is capable of more accurate facial action unit recognition and real-time performances.

**TABLE 2:** Action units provided by Openface 2.0.

AU code	Description
AU 01	Inner brow raiser
AU 02	Outer brow raiser
AU 04	Brow lowerer
AU 05	Upper lid raiser
AU 06	Cheek raiser
AU 07	Lid tightener
AU 09	Nose wrinkle
AU 10	Upper lip raiser
AU 12	Lip corner puller
AU 14	Dimpler
AU 15	Lip corner depressor
AU 17	Chin raiser
AU 20	Lip stretcher
AU 23	Lip tightener
AU 25	Lips part
AU 26	Jaw drop
AU 45	Blink

**FIGURE 3:**
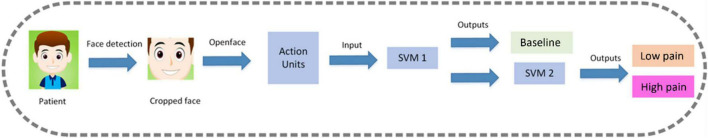
The proposed classification tree for the three pain levels.

Support Vector Machines (SVMs) were employed for classification. SVMs have great performance in classification since they can map the training examples onto a higher dimensional space and then determine the optimal separate hyperplane based on Structural Risk Minimization theory. The extracted AUs are chosen as the input of the SVMs. To simplify the work, all data was partitioned into three classes: baseline (B), low pain (LP), and high pain (HP). The data labeled with ratings from 1 to 5 were categorized as low pain. The data labeled with ratings from 6 to 10 were categorized as high pain. A classification tree was tried to perform the multi-classification task. We use SVM 1 to classify the pain vs. no pain. Then SVM 2 is used to classify low pain vs. high pain. The proposed classification tree is shown in Figure 3.

The EEG data were filtered with a 1–50 Hz band-pass filter. Independent Components Analysis (ICA) was used to remove the artifacts from the raw EEG signal. Fast Fourier Transformation (FFT) was used to convert the raw EEG signal from time-domain to frequency domain. The pupillary diameter data was filtered with the “pupillary velocity” method (Bergamin et al., 1998).

MATLAB and Python are used to analyze the data. For facial expression images, Openface 2.0 is utilized as the feature extractor and a cascade of SVMs is developed to perform the classification. For the physiological data, an analysis of variance (ANOVA) is used to test the null hypothesis (*p* < 0.05).

## Results

### Subjective Ratings

**FIGURE 4:**
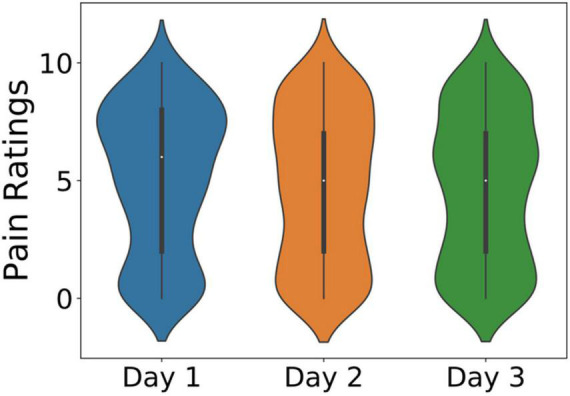
The subjective ratings from three repeated days of all the subjects.

The subjects’ pain level is recorded every 20 s during the experiment using the VRS in three repeated experiments in three different days. The experiment is repeated three times for each participant to investigate whether the participants get acclimatized to the cold pain stimulus. Figure 4 shows three violin plots of the subjective ratings of all the subjects on three different days. Day 1, day 2, and day 3 for each subject were mostly from the same day in three consecutive weeks. The violin plot of day 1 was wider in the upper part, which meant there were more high pain scores on day 1 than on day 2 and day 3, according to the shape of the violin plots.

### Facial Expression

The response to a person’s internal emotional states, intentions or inter-personal communications can alter his/her facial expression. Pain measurements can be gathered using computer-based analysis and pattern recognition software that captures facial features and their changes.

In this section, the performance of the proposed classification tree for the three-level pain intensity estimation is evaluated. F1 score is used as the criterion for the classification performance. We first use the 5-folds cross-validation strategy, where all video frames are randomly partitioned into five equal-sized subsample sets; four of the five sets are selected as the training set and the remaining one is selected as the testing set. The whole process is repeated five times and then the averaged F1 score is computed as the final performance outcome. The results are shown in Table 3. The best performance is achieved at baseline level, with an F1 score of 73.25%. To further investigate the generalization of our proposed classification tree model, we also use to leave one subject out validation. In this process, frames from one subject are selected as the testing set and the remaining frames are selected as the training set. The whole process is repeated 29 times since there are 29 subjects taking the experiment, and the final outcome will be the averaged F1 score as well. The best results are achieved at baseline level with an F1 score of 58.62%.

**TABLE 3:** The results of the two validation strategies for the pain intensity estimation.

Pain intensity	5-folds cross validation (F1-score %)	Leave one subject out validation (F1 score %)
Baseline	73.25	58.62
Low pain	61.67	47.71
High pain	69.83	51.39

### Electroencephalography

**FIGURE 5:**
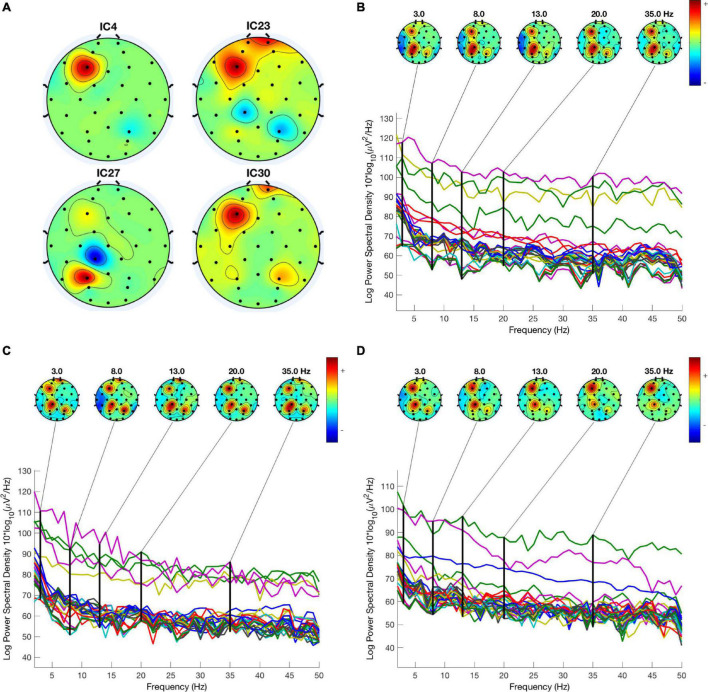
**(A)** Four common artifacts detected by the ICA. **(B–D)** EEG spectrum and topography in baseline, low pain, and high pain states.

Electroencephalography is the measurement of electricity generated by the brain. The sensor system used to measure the activation signal includes electrodes that follow the International 10–20 system, as shown in Figure 2. After screening through a band-pass filter, the EEG data is converted from time-domain to frequency domain by using FFT. There are frequency bands over the EEG frequency, namely delta (1–3 Hz), theta (4–8 Hz), alpha (8–13 Hz), beta (13–30 Hz), and gamma (30–50 Hz). Figure 5A shows the topographies of common EEG artifacts, namely generic discontinuities (GD), eye blinks (EB), horizontal eye movement (HEM), and vertical eye movement (VEM). Figures 5B–D shows the spectral plot and topography of the EEG signal in baseline, low pain, and high pain states. The EEG spectral power increased around the Parietal area over all EEG frequency bands (dof = 25, *p* < 0.01). The EEG spectral power decreased around the Central-Parietal area in alpha, beta, and gamma bands (dof = 25, *p* < 0.01).

### Eye Movement

**FIGURE 6:**
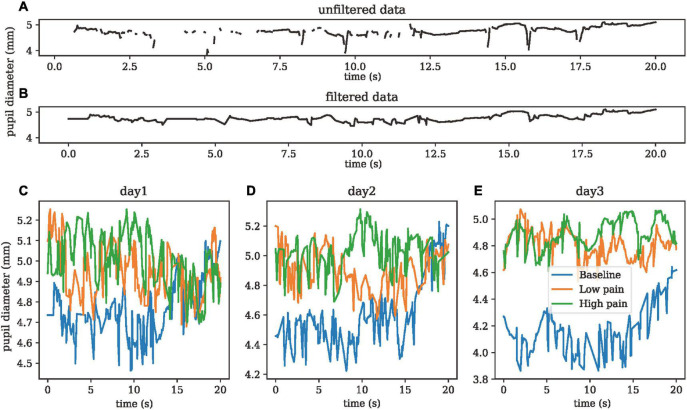
**(A)** Un-filtered pupil diameter data. **(B)** Filter pupil diameter data. **(C–E)** Pupil diameter data of one subject in three painful states on day 1, day 2, and day 3.

Eye movement and pupillary unrest can both be observed in objective pain measurement studies as the human eye will react when pain or discomfort is felt. Such reactions are due to closing of the eyes, eye movement, or pupillary unrest. The eye movement measurement system is among the simplest, as users will wear a pair of Tobii Eyeglasses and remain still with their eyes open. The Tobii eye tracker is designed as a pair of glasses, with open space in place of lenses, and is applied as a wearable sensor over the face. Users that wear eyeglasses are allowed to wear the Tobii Eyeglasses over their eyeglasses if preferred. Figures 6A,B show the unfiltered and filtered pupil diameter data (mm), respectively. Figures 6C–E show the pupil diameter of one subject in baseline, low pain, and high pain states in three different days. ANOVA showed significant differences between three pain states for pupil diameter (dof = 25, *p* < 0.01).

### Skin Conductance

**FIGURE 7:**
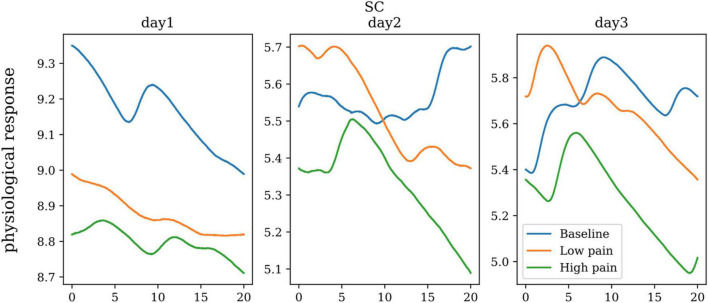
The skin conductance of one subject on three repeated days.

Skin Conductance (SC) is the measurement of electricity conducted by the skin. A small amount of voltage is applied through two electrodes that are typically strapped to two fingers of one hand, providing real-time variation in conductance. Since skin conductance is a non-intrusive, easy-to-measure signal. It can also be timesaving as skin conductance bio-sensors are applied externally and can immediately gather data. Figure 7 shows the skin conductance of one subject in baseline, low pain, and high pain states in three different days. ANOVA showed significant differences between the three pain states for skin conductance (dof = 25, *p* < 0.01).

### Blood Volume Pulse

**FIGURE 8:**
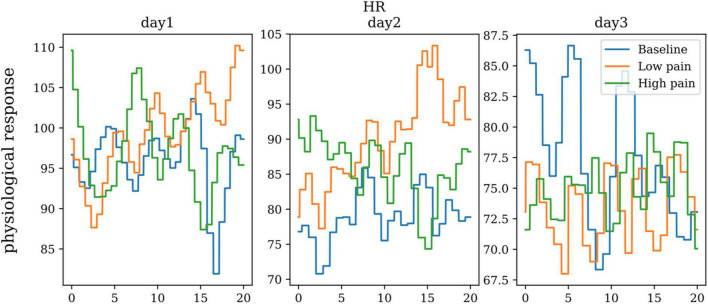
The heart rate of one subject on three repeated days.

Blood Volume Pulse (BVP) bounces infrared light against a skin surface and measures the amount of light reflected. The amount of light reflected varies with the amount of blood that is present in the skin – vasomotor activity and sympathetic arousal. The peak-to-peak amplitude of the signal will vary with respect to the changes in sympathetic arousal. Figure 8 shows the heart rate of one subject in baseline, low pain, and high pain states in three different days. ANOVA did not show any significant differences between the three pain states for heart rate.

### Electromyography

**FIGURE 9:**
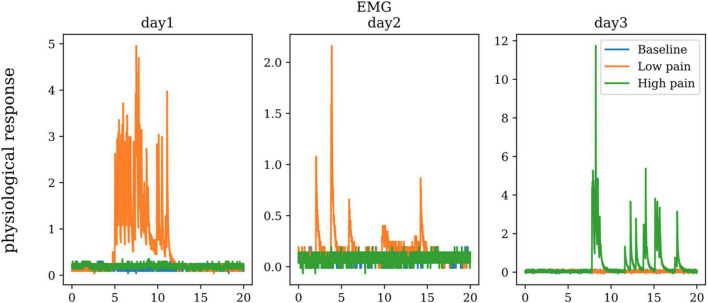
The electromyography (EMG) of one subject on three repeated days.

Electromyography is the measurement of muscle activity accomplished by detecting and amplifying the tiny electrical impulses that are generated as the muscle fibers contract. The electromyograph is the sensor used to measure the activation signal of muscles. This sensor is placed on the muscle belly with positive and negative electrodes parallel to the muscle fibers. Depending on the muscles under observation, the measured EMG potentials can range between less than 50 microvolts to within 20–30 mV. There are two measurements of EMG signals: (1) Needle (intramuscular) EMG, and (2) Surface EMG. Our study uses on the Surface EMG because it is the least intrusive. Figure 9 shows the EMG of one subject in baseline, low pain, and high pain states during three different days. ANOVA didn’t show any significant differences between the three pain states for EMG.

### Respiration Rate

**FIGURE 10:**
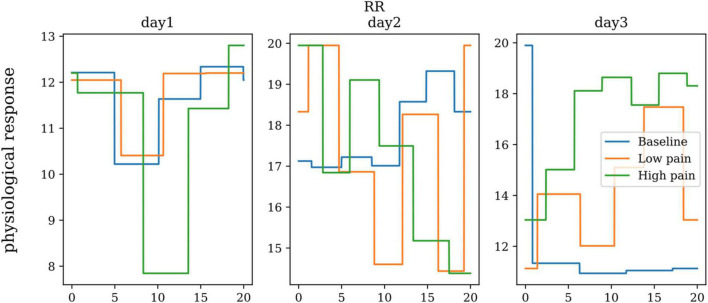
The respiration rate of one subject on three repeated days.

The respiration sensor is an elastic medium that is sensitive to stretch. This sensor is strapped around the subject’s chest or abdomen. It converts contraction and expansion of the rib cage of the abdominal region – causing rise and fall of the signal. When a person is in pain, breathing rhythms change. This rhythm can be controlled where the person in pain can try to find a relaxed state of mind to heal, or it can be less controlled, leading to a state of panic. Breathing exercises can be implemented to relieve and/or cope with pain. Figure 10 shows the respiration rate from one subject in baseline, low pain, and high pain states in three different days. ANOVA didn’t show any significant differences between the three pain states for respiration rate.

### Skin Temperature

**FIGURE 11:**
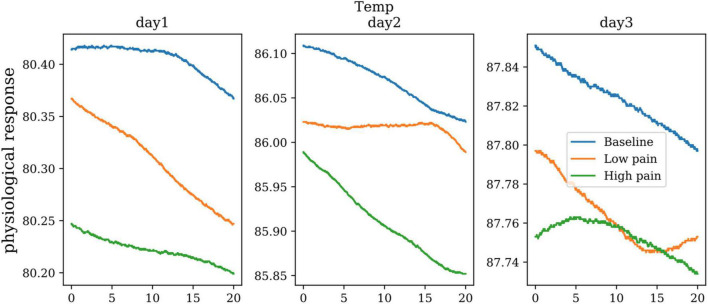
The skin temperature of one subject on three repeated days.

The body’s peripheral temperature, as measured on its extremities, will vary according to the amount of blood perfusing the skin. This, in turn, is dependent on the subject’s state of sympathetic arousal. Changes in temperature during pain varies by person. There is no standard threshold for every human test subject; however, the changes in temperature vs. time during a painful situation can provide an insight into increasing and decreasing pain levels. Figure 11 shows the skin conductance from one subject in baseline, low pain, and high pain states in three different days. ANOVA showed significant differences between three pain states for skin conductance (dof = 25, *p* < 0.01).

### Blood Pressure

**FIGURE 12:**
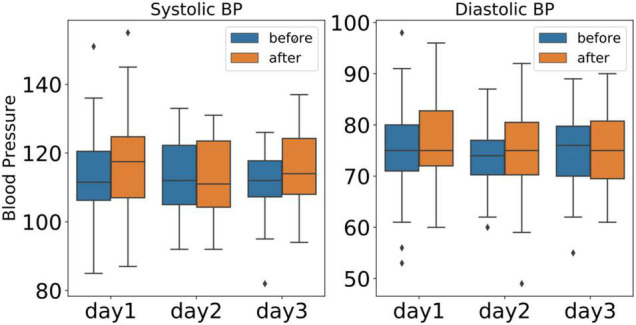
The boxplots of systolic and diastolic blood pressure from all the subjects over the course of 3 days (blue: before cold pressor test, orange: after cold pain test).

Variation in systemic blood pressure can be a manifestation of pain response. Blood pressure sensors are typically non-invasive as they are designed to measure systolic, diastolic, and mean arterial pressure via the subject’s oscillating blood pressure responses. Figure 12 shows the boxplots of the systolic and diastolic blood pressure from all subjects over the course of 3 days. For systolic blood pressure, there was a significant increase after the cold pain test (dof = 25, *p* < 0.01).

### Multi-Signals Fusion at Decision Level

**FIGURE 13:**
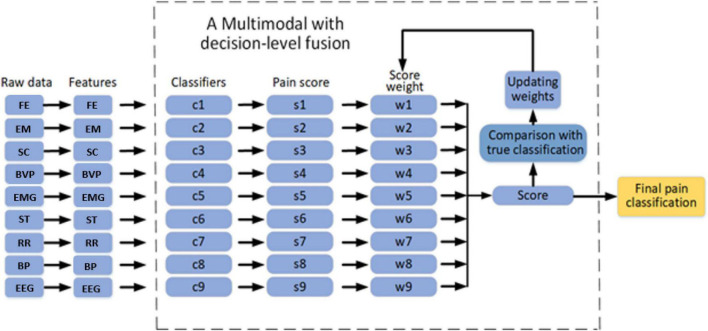
The flowchart of our decision-level multi-modal fusion [facial images (FE), electroencephalography (EEG), eye movement (EM), skin conductance (SC), and blood volume pulse (BVP), electromyography, respiration rate (RR), skin temperature (ST), and blood pressure (BP)].


Based on the above results on individual signal, we came up with a multi-signal based pain sensing system as shown in Figure 13, the flowchart of our multi-modal with decision-level fusion. The Genetic algorithm (GA) (Mirjalili, 2019) was applied in the Multi-signals fusion part. The procedure of the fusion is as follows. The fused score can be defined using the formula:

(1) $$f\,s=w_{1}\,s_{1}+w_{1}\,s_{1}+\ldots +w_{9}\,s_{9}$$

where *w*_i_ represents the weights and *s*_i_ represents the score achieved from the sensor *i*, for *i* = 1,2,…,9. If the difference between the true score and fused score is more than a specified threshold, the weights are updated by GA until the stopping criteria is satisfied. GA is widely used as evolutionary computation technique. A candidate in GA is called an individual or a chromosome, which contains a set of solutions. Acting as the biological evolution, GA begins searching from a randomly generated chromosomes and continues to select fitter chromosomes during each generation. The population which consists of the weights *w*_i_ are initialized. Then, the set will go through the selection, crossover, and mutation processes of the GA. In the selection part, fittest individuals are selected and generated by the GA. In the crossover part, features of good surviving individuals are propagated into the next population, which will have a better fitness than the current population. The mutation process promotes diversity in population and keeps the GA from getting stuck in local search. In the manuscript, the crossover rate and mutation rate were set to 0.8 and 0.02, respectively. The initial population size and maximum iterations were set to 50.

**FIGURE 14:**
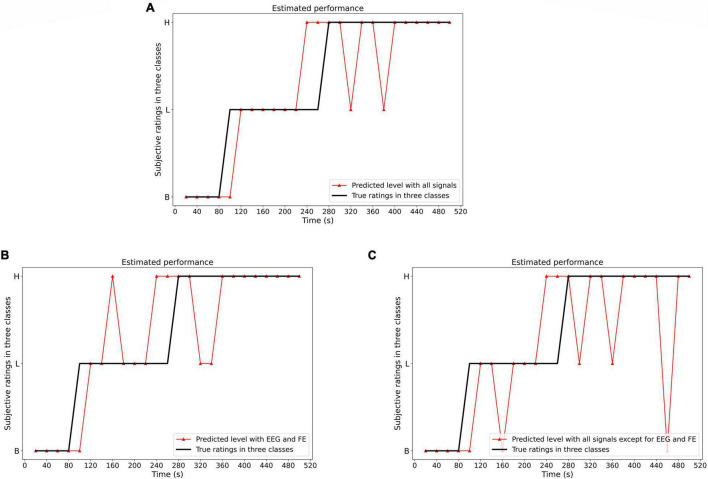
The classification result using different sets of modalities (set 1: all signals; set 2: only EEG and FE; set 3: all signals but EEG and FE). The estimated performance with all signals performs better than that without EEG and FE, especially in Baseline and High pain level estimation. The second set with only EEG and FE performs better than that without EEG and FE, especially in High pain level estimation. **(A)** Set 1: Estimation result (all signals are used, B, baseline; L, low pain; H, high pain). **(B)** Set 2: Estimation result (only EEG and FE are used, B, baseline; L, low pain; H, high pain). **(C)** Set 3: Estimation result (all signals are used except EEG and FE. B, baseline; L, low pain; H, high pain).

The multi-modal is tested using different sets of parameters. Figure 14 shows the classification performance of our dataset using two sets of physiological modalities. The first set contains all signals. The second set only contains EEG and FE. The third set includes all signals but EEG and FE. When EEG and FE are removed from the modalities, the performance of the pain intensity estimation becomes worse than that of using all the modalities, especially in the low pain and high pain intensity estimation. When only EEG and FE are used, although it performs worse than the first set that used all signals, but still performs better than the third set where EEG and FE are removed, especially in the high pain intensity estimation. The third set estimated one of the high pain level into baseline which didn’t occur in the second set. This shows that EEG and FE have an immediate impact on the pain intensity estimation.

## Discussion

### Physiological Signals

In the facial expression based cold pain intensity estimation task, we employed a cascade SVM based classification tree and utilized the Action Unit (AU) regression values as the input. Two validation strategies are applied to estimate the performance of the proposed model. The results suggest that the cold pain intensity estimation based on facial expression is a feasible task. The best estimation performance is achieved at no pain level. We also found that the outcome of the 5-fold cross-validation is better than that of the one-subject-left-out validation, which indicate that pain perception is individually different. In future work, more data will be collected to enhance the performance of the model. In addition, in this study we used all AU information from the Openface software, which may have some redundancies among them. We found that, among all AUs, the AU45, which represents the eye blinks, has the least effect on the estimation between pain and no pain status. However, AU4, AU9, and AU10 had the most effect. A direction for the next steps is to investigate the best combination of the most useful AUs and remove the redundant ones to better estimate the cold pain intensity. Another step is to investigate the connection between AU intensities and pain intensities and get the best combination of AUs to estimate the cold pain intensity.

ANOVA showed that there are significant differences between the pupil diameter measurement in three painful states. Pupillary unrest can also provide a clear response to pain. Bokoch et al. (2015) showed that pupillary unrest in the presence of ambient light (PUAL) is a disordered changeability that varies with light intensity. In assessing pain using the variation coefficient of pupillary diameter, Charier et al. (2017) explained that the sympathetic and parasympathetic nervous systems interact within the iris. They evaluated the basic mean pupillary diameter, how it increased during a contraction, and pain via the NRS. Using a portable video pupillometer, they were able to record the pupillary diameter (PD) with 0.05 mm accuracy. They concluded that there is clinical relevance of variation coefficient of PD (VCPD). More accurate pain assessment can be carried out on those who are unable to communicate.

In the present study, the EEG spectral power increased around the Parietal area over all EEG frequency bands. Lutzenberger et al. (1997) showed the topographic map of the dimensional complexity of the EEG during acute pain, personal pain, and personal stress. This study was conducted by the authors to test the associative networks between the cortical cell assemblies representing pain-related memories being more vivid in subjects with chronic pain. A study by Rissacher et al. (2007) further showed how the EEG can detect strong activity in the brain. In their study of identifying frequency-domain features for an EEG-based pain measurement system, they showed how information in the frequency domain from EEG can provide features of pattern recognition to observe pain characteristics. Using human test subjects during pain and control trials, they concluded that the temporal-parietal decrease in Alpha may index the activation of brain areas during pain.

ANOVA showed there were significant differences between the skin conductance measurements in three painful states. For pain assessment, attaching adherent electrodes to the subject’s palm or hand or sole of the foot is convenient and effective. A fast-responding conductance signal is then processed, and the frequency of fluctuations was converted into a measured unit (Cowen et al., 2015). The signal was thought to be independent of adrenergic agents, hemodynamic variability, and respiratory rate, as sweat glands are controlled by the muscarinic receptors. Due to the skin quality, moisture levels, and environmental temperature, the signal could not be pain-specific (Cowen et al., 2015). Although the signal may not be pain-specific, there is a need to differentiate between pain and discomfort. Furthermore, skin conductance can be used to study what is being “felt” by patients objectively.

Skin temperature data showed significant differences in three painful states. Kammers et al. (2011) also showed the important connection between pain, regulation of body temperature, and body ownership. The authors investigated whether external manipulation of body temperature could influence body ownership and concluded that the painful extremes of the varying temperatures do not modulate the RHI. Skin temperature varies based on the body’s health conditions and time of day, as well as factors such as pain, illness, and nervous tension. Tse et al. (2016) studied the accuracy of a hand-held infrared device for estimating peripheral skin temperature and the detection of temperature disparities, and infrared devices were useful tools for this purpose.

Blood pressure is another promising modality for pain detection. There is clear evidence showing that blood pressure readings vary with pain intensity. Scheuren et al. (2016) explained that the numerical rating scale can be used to quantify subjective pain intensity and pain unpleasantness. Subjects were classified as responders and non-responders to thermal grill stimulation based on pain intensity ratings. Responders exhibited lower systolic and diastolic blood pressure than non-responders and inverse linear association is observed between blood pressure and pain intensity and unpleasantness.

### Future Research Directions

Based on the strengths and limitations of the current model and performances, we envision the following directions guiding the next step of the research.

#### Multi-Modal Pain Recognition System

As pain is a continuous sensation with no fixed point of reference, ongoing research continues to grow toward developing systems that can continuously measure pain. Multi-modal machine learning is a fast-growing field in this research area. Pain measurements can also be gathered using computer- We will continue our efforts in the direction of multi-modal pain recognition.

#### Pain Remote Assessment in the COVID

Novel coronavirus COVID-19 is spreading and is an urgent threat that has sped up a shift from the traditional in-person diagnosis to the remote assessments in many healthcare fields, including pain intensity assessment. In order to objectively diagnose a patient’s current pain intensity and fully protect healthcare workers, remotely assessing pain intensity is a good alternative to replace in-hospital diagnosis. Specifically, one future research direction is to design a home-use pain diagnosis software for patients and/or caregivers. This software works as a personal pain diagnosis assistant for at-home use by patients to replace unnecessary in-hospital visits and provide accurate pain records in the past periods. It would connect with wireless wearable sensors, monitor the patient’s physiological signals, estimate the patient’s current pain intensity scores, and periodically send historical records to the patient’s healthcare workers or pain doctors.

#### Multidimensional Pain Representation

A one-dimensional scale on pain intensity lacks the possibility to capture the complexity of the patient’s pain experience. In our project, using one-dimension to represent the pain experience could also be responsible for the limitations of the algorithms’ performances. Hence, discovering a different way to represent and quantify the patients’ overall pain experience is one of the future research directions. Multiple pain assessment tools have been developed to represent the pain experience in a multifaceted way, even though there is no consensus on multiple dimension pain assessment in clinical use. For example, Clinically Aligned Pain Assessment (CAPA) Tool (Topham and Drew, 2017) measures pain in five dimensions, including comfort, change in pain, pain control, functioning, and sleep quality. Brief Pain Inventory (BPI) Multidimensional tool (Cleeland and Ryan, 1994) evaluates pain experience in six dimensions, such as pain intensity, sleep, walking ability, mood, relations with others, and ability to concentrate. Another commonly used assessment tool, Initiative on Methods, Measurements, and Pain Assessment in Clinical Trials (IMMPACT) (Dworkin et al., 2005) designed six outcome domains for the chronic pain clinical trials. They are pain intensity, physical functioning, emotional functioning, participant ratings of improvement and satisfaction, symptoms and adverse events, and participant dispositions.

Summarizing the current multidimensional pain intensity tools and considering the functioning of the PC and mobile platforms, the pain representation should minimize the subjective impact on the dimension measuring and maximize the dimensions to evaluate the subject’s pain experience. Therefore, one possible multidimensional pain representation is the six-dimension: pain intensity and change in pain, sleep, functioning and walking ability, comfort and mood, the ability to concentrate, and the patient’s disposition. Mobile and wearable digital devices can monitor and evaluate the patient’s performance in these six dimensions, either by subjectively entering the ratings, or objectively measuring their current states in different dimensions. For example, the sleep function and the step function in the smart watch can monitor the subject’s sleeping quality and walking capability objectively, and the front camera can predict the subject’s current moods and emotions. However, these six dimensions are not independent of each other but exist correlations. These correlations, such as sleep, pain and mood, can lead to different treatments and different dimensions. These relationships will be investigated more thoroughly in our future endeavors.

## Conclusion

In this work, we conducted an experimental study using nine physiological modalities: facial images, electroencephalography, pupillary diameter, skin conductance, blood volume pulse, electromyography, respiration rate, skin temperature, and blood pressure. Machine learning algorithms and ANOVA were used to analyze the physiological data and features. The results indicated that facial expressions, eye movement, EEG, skin conductance, skin temperature, and blood pressure proved to be the most promising to detect the different levels of painful states. A decision-level multi-modal fusion has potential to improve the accuracy of classifying painful states.

## Data Availability Statement

The raw data supporting the conclusions of this article will be made available by the authors, without undue reservation.

## Ethics Statement

The studies involving human participants were reviewed and approved by the Institutional Review Board of Northeastern University (IRB #17-01-25). The patients/participants provided their written informed consent to participate in this study. Written informed consent was obtained from the individual(s) for the publication of any potentially identifiable images or data included in this article.

## Author Contributions

YL, YX, and RU conceived the conception and designed of this research. LW, YG, and WZ executed the experiments, including data acquisition, and analysis. LW, YG, WZ, BD, and YL drafted the manuscript. YL, YX, RU, SK, KS, and RE revised the manuscript. All authors contributed to the article and approved the submitted version.

## Conflict of Interest

The authors declare that this study received funding from National Science Foundation #1838796, 1838650, and 1838621. RU reports fees and/or research funding from Merck, Covidien/Medtronic, AcelRx, Pfizer, NIH, and AHRQ. YX reports research funding from AHRQ. YL reports research funding from NSF. All the funders were not involved in the study design, collection, analysis, interpretation of data, the writing of this article or the decision to submit it for publication.

## Publisher’s Note

All claims expressed in this article are solely those of the authors and do not necessarily represent those of their affiliated organizations, or those of the publisher, the editors and the reviewers. Any product that may be evaluated in this article, or claim that may be made by its manufacturer, is not guaranteed or endorsed by the publisher.
